# 592 Trends in First Positive Culture Results in Major Burn Center over a 10-year Period

**DOI:** 10.1093/jbcr/iraf019.221

**Published:** 2025-04-01

**Authors:** Gabrielle Bierlein-De La Rosa, Shady Al Hayek, Colette Galet, Patrick Ten Eyck

**Affiliations:** University of Iowa; University of Iowa; University of Iowa; Institute for Clinical and Translational Science - University of Iowa

## Abstract

**Introduction:**

Burn wounds are unique, yet common injuries that damage the cutaneous barrier, increasing risk of infection. Patients with severe burns, defined as total burn surface area (TBSA) >20% are more vulnerable to infection which may lead to sepsis and death. With the heavy use of topical and systemic antimicrobials, changes in the burn wound microbial and antibiotic resistance patterns have been reported; however, the literature remains scarce. This retrospective cohort study was designed to assess wound infection trends in first positive cultures in our burn unit over the last 10 years.

**Methods:**

This is a retrospective cohort study. Our burn registry was queried to retrieve all patients admitted to our burn unit from July 2013 to June 2023. Demographics, TBSA, injury and admission information were obtained from the burn registry. First positive wound culture information including dates of first positive cultures, gram positive, gram negative, and fungal organisms were collected from medical records. Patients were stratified based on TBSA as small (< 10% TBSA), moderate (10-19.9% TBSA), and severe burns (>20% TBSA). Descriptive statistics were obtained. Generalized linear models were fit to assess the trends of positive cultures and length of hospitalization over time for the three TBSA strata.

**Results:**

A total of 2,755 adult and pediatric patients were included; median age was 38 years, 72.2% were male, 74.1% presented with small burns, 15.9% with moderate burns, and 10.1% with severe burns. Wound cultures were performed in 40.3% of our population with 600 having positive first cultures; 84.7% grew Gram +, 35.7% Gram-, and 9.7% fungal organisms. Rates of Gram – species increased annually in first positive cultures by 6.6% in severe burns on average (Relative rate (RR) = 1.066 [1.026-1.107]). Fungal infections also increased annually in severe burns by 15.1% on average (RR = 1.151 [1.046-1.267]). Gram + species, on the other hand, decreased by 6.6% annually in severe burns, on average (RR = 0.934 [0.910-0.958]).

**Conclusions:**

Our data showed differing trends in varying microorganisms over the study period in patients with severe burns. Future studies are warranted looking at the relationship between infection rates and antibiotic resistance.

**Applicability of Research to Practice:**

Regular monitoring of bacterial trends in the burn unit would be helpful for choosing prophylactic antibiotics for burn infections, especially for severe burns.

**Funding for the Study:**

National Center For Advancing Translational Sciences of the National Institutes of Health Award Number UM1TR004403.

